# Coronary sequelae of Kawasaki disease treated with rotational atherectomy and drug coated balloon: A case report

**DOI:** 10.1097/MD.0000000000018371

**Published:** 2020-01-03

**Authors:** Yongfeng Shi, Longbo Li, Meijia Liu, Chunli Song, Junduo Wu, Bin Liu

**Affiliations:** Cardiology Department, The Second Hospital, Jilin University, Changchun, Jinlin, 130041, PR China.

**Keywords:** artery aneurysm, drug coated balloon, Kawasaki disease, rotational atherectomy

## Abstract

**Introduction:**

Kawasaki disease (KD) is an acute vasculitis syndrome that mainly affects children and is the first cause of acquired heart disease. Coronary artery lesion is the most serious complication of KD. Only two previous studies have reported similar cases, but we reported patient was younger and had a longer follow-up.

**Patient concerns:**

We reported a case of coronary sequelae of KD treated with rotational atherectomy and drug coated balloon (DCB). During the week after surgery, the patient complained of a slight chest pain intermittently, but no longer appeared after that.

**Diagnosis:**

We diagnosed by electrocardiogram and angiography. Angiography showed that the anterior descending branch (LAD) proximal stenosis was 95%, the right coronary artery (RCA) middle stenosis was 99%, and the calcification was severe.

**Interventions:**

We treat the patient with rotational atherectomy using a 1.25 mm burr, pre-dilatation of the stenosis lesion with a 3.5 mm × 15 mm non-compliant balloon was achieved. Then 3.5 mm × 15 mm drug eluting balloon was inflated at 10 atm for 60 seconds.

**Outcomes:**

After the 6-month follow-up (from October 2018 to March 2019), the symptom of angina disappeared. Coronary angiography 6 months later showed no apparent progression of vessel narrowing.

**Conclusion:**

The present case suggests that rotational atherectomy followed by DCB dilation could be an alternative revascularization therapy of choice in coronary KD sequelae complicated with atherosclerosis.

## 1 Introduction

Coronary sequelae of Kawasaki disease (KD) is the most common cause of acquired heart disease in childhood, and the KD patients are fatal due to acute thrombosis and occlusion.^[1]^ It has a variety of clinical manifestations, such as aneurysm or ectasia, stenosis, and severe calcification.^[2]^ Surgical bypass is the main treatment for complex coronary artery sequelae of KD.^[3]^ However, it is difficult to treat KD by percutaneous coronary intervention (PCI), because it causes thrombosis and the formation of advanced aortic aneurysms. Herein, we present a patient with complex coronary sequelae of KD treated with rotational atherectomy and drug coated balloon (DCB) and good follow-up results.

## 2 Case report

**Figure 1:**
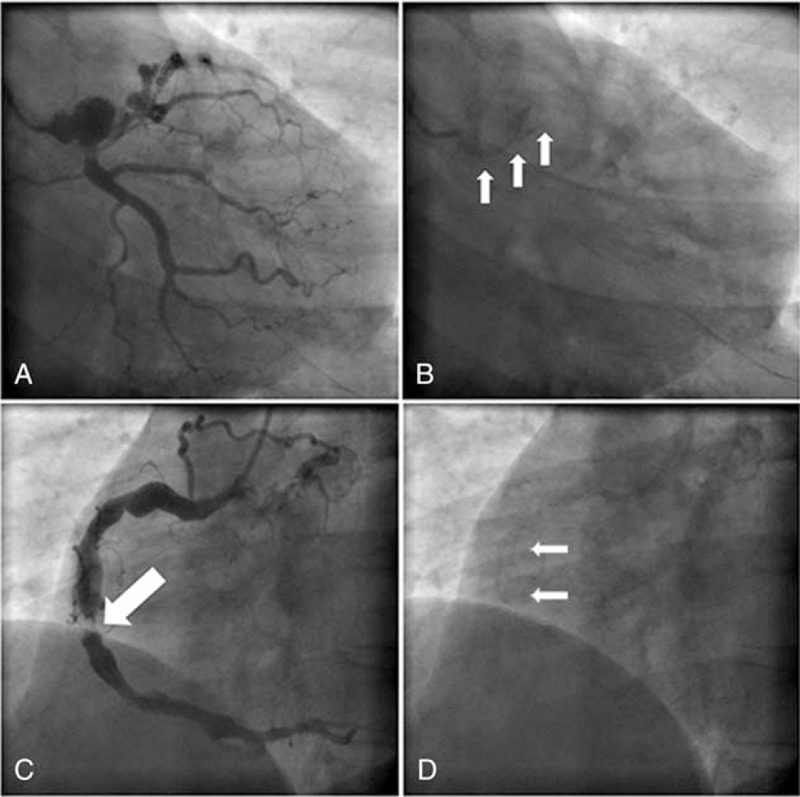
Coronary angiography. A, Giant artery aneurysm (15.68 mm × 12.59 mm) between LM and LAD. The proximal of LAD is nearly occluded; B, Severe calcification at the site of giant artery aneurysm (white arrow); C, Artery aneurysm located from proximal to distal RCA (the maximal width is 8.23 mm). 99% stenosis in the middle of RCA (white arrow); D, Server ecalcification at the site of stenosis (white arrow). LAD = anterior descending branch, LM = left main, RCA = right coronary artery.

A 35-year-old male with KD visited our outpatient department. He told us that he had experienced chest pain in the past 8 days, and electrocardiograph (ECG) showed that Q wave in ECG leads II, III, and aVF. He was treated with 300 mg aspirin and 300 mg clopidogrel, followed by 100 mg aspirin and 75 mg clopidogrel, respectively daily. Angiography revealed (Fig. 1) giant artery aneurysm between left main (LM) and anterior descending branch (LAD), with 95% stenosis in the proximal of LAD and severe calcification. An artery aneurysm was located from proximal to distal right coronary artery (RCA), and with 99% stenosis and severe calcification in the middle of RCA. There were collaterals from RCA to LAD.

**Figure 2:**
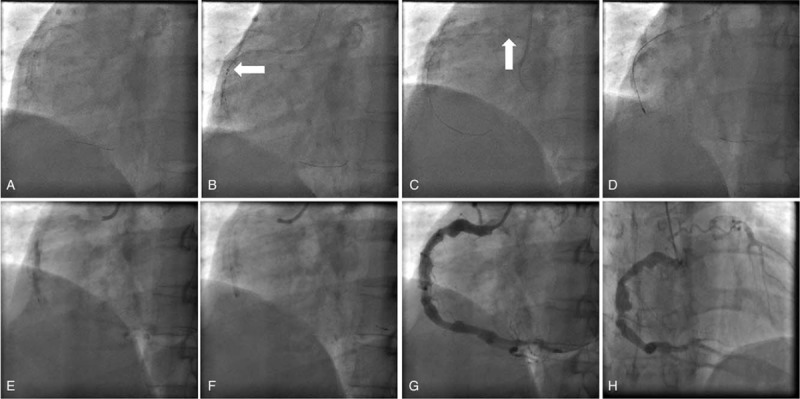
Operation procedures. A, 2.5 mm × 15 mm balloon cannot pass through the culprit lesion; B, Guidezilla was deep-seated into the middle of RCA (white arrow: the tip of guidezilla); C, Guidezilla was pushed away from the ostium of RCA (white arrow: the tip of guidezilla); D, Rotational atherectomy with a 1.25 mm burr in the middle of RCA; E, Pre-dilatation with a 3.5 mm × 15 mm non-compliant balloon; F, 3.5 mm × 15 mm DCB; G, The final result; H, Angiography 6 months later. DCB = drug coated balloon, RCA = right coronary artery.

**Figure 3:**
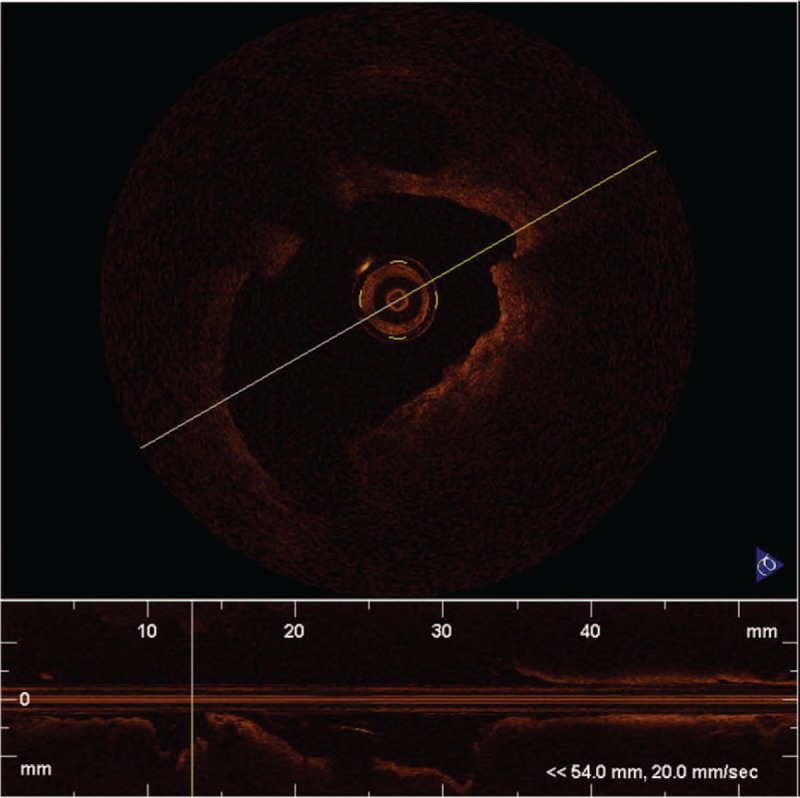
Optical frequency domain imaging images 6 months later (minimum lumen area: 5.93 mm^2^).

A Launcher Coronary Guiding Catheter (6F JR 4) (Fig. 2) was placed in the opening of the right coronary artery. A 0.014-in. run-through guidewire was advanced to the distal of RCA. Firstly, we failed on pre-dilatation of the lesion with a 2.5 mm × 15 mm balloon due to lack of supporting. And then guidezilla was deep-seated into the middle of RCA, and we used 1.5 mm × 15 mm balloon, but failed again. After rotational atherectomy using a 1.25 mm burr, pre-dilatation of the stenosis lesion with a 3.5 mm × 15 mm non-compliant balloon was achieved. Then 3.5 mm × 15 mm drug eluting balloon was inflated at 10 atm for 60 seconds. There were no complications during balloons inflations and the result looks good. In addition, during the 6-month follow-up, the symptom of angina disappeared and the follow-up angiography showed a good result (Fig. 3). Patient has provided informed consent for publication of the case.

## 3 Discussion

Kawasaki disease, firstly being described by Tomisaki Kawasaki in 1967,^[4]^ is a systemic vasculitis, and is the most common cause of acquired heart disease in childhood. Usually it is self-limiting but sometimes it can be fatal because of coronary artery sequelae.^[2]^

Coronary sequelae of KD has a variety of clinical manifestations, such as aneurysm or ectasia, stenosis, and severe calcification.^[2]^ Coronary artery aneurysm is defined as the dilation of the coronary artery measuring 1.5 times the diameter of a normal adjacent segment and noticed in approximately 15% to 25% of the children with KD.^[5]^ The proximal end of the LAD part is the predilection site of coronary artery aneurysms.^[6]^ According to the American Heart Association statement, aneurysms were classified as small (<5-mm internal diameter), medium (5–8 mm internal diameter), or giant (>8-mm internal diameter).^[7]^ 30% to 50% of such aneurysms will regress but the others will remain the same or become stenotic or calcification, leading to thrombosis and ischemic heart disease.^[8]^ Ming-Tai Lin et al reported that myocardial ischaemia, AMI, or death occurred in 7.1% of these patients.

Surgical bypass is the main treatment for complex coronary artery sequelae of KD, but nowadays several case reports reveal that stent implantation is feasible for these patients.^[3,9–11]^ However, long-term outcome of stent implantation in these groups is still unknown. The diameter of culprit lesions is hard to measure because of artery ectasia, always result in underestimation of diameter and even stent malposition. High-pressure balloon dilatation is always needed because of dense fibrosis and heavy calcification of the culprit lesion, which can lead to late neo-aneurysmal formation in KD patients.^[10,11]^ And the stiff lesion can result in stent under expansion and put the patients at the risk of acute thrombosis.^[12]^

Rotational atherectomy has become a strategy for local coronary stenosis combined with heavy calcification in KD patients. And the use of a larger burr can produce good patency with close follow-up.^[7]^ Restenosis within the first year after PTCRA often develops because of reactive intimal thickening.^[13]^ Drug coated balloon is a new way to reduce the rate of restenosis. So rotational atherectomy followed by DCB dilation is an ideal method to treat coronary stenosis of KD.

Stent implantation for coronary stenosis after KD is difficult. In this case, CA shows aneurysm, stenosis, and severe calcification. The balloon could not pass through the culprit lesion because of severe calcification. Even with the help of guidezilla catheter deep-seated into the middle of RCA, we still failed on balloon delivery. Finally, after PTCRA using a 1.25 mm burr, pre-dilatation of the stenosis lesion with a 3.5 mm × 15 mm non-compliant balloon was achieved. In order to reduce the rate of restenosis and avoid the risk of acute thrombosis, we used a 3.5 mm × 15 mm drug coated balloon instead of stent implantation. And 6 months later, the follow-up angiography showed no restenosis at the culprit lesion. Follow-up OCT showed no reactive intimal thickening or late neo-aneurysmal formation and clinical follow-up showed that the symptom of angina disappeared.

In the present study, we introduced a new and feasible treatment program for coronary sequelae of Kawasaki disease, atherectomy, and drug-coated balloons. It could provide a reference for the treatment of coronary sequelae of Kawasaki disease, but it should be pointed out that we had not conducted large-scale clinical trials and long-term follow-up.

## Author contributions

**Data curation:** Yongfeng Shi, longbo Li, Junduo Wu.

**Formal analysis:** Yongfeng Shi, Chunli Song.

**Project administration:** Bin Liu.

**Writing – Original Draft:** Yongfeng Shi, Meijia Liu, Bin Liu.

**Writing – Review & Editing:** Yongfeng Shi, Bin Liu.
